# Climate change and mental health in the Philippines

**DOI:** 10.1192/bji.2022.31

**Published:** 2023-05

**Authors:** Rowalt Carpo Alibudbud

**Affiliations:** Assistant Professorial Lecturer, Department of Sociology and Behavioral Sciences, De La Salle University, Manila, Philippines. Email: rowalt.alibudbud@dlsu.edu.ph

**Keywords:** Climate change, mental health, Philippines, Typhoon Haiyan, Super Typhoon Yolanda

## Abstract

The mental health repercussions of the climate crisis are observed annually in the Philippines, one of the world's most climate-vulnerable countries. This paper explores these repercussions by examining the aftermath of Typhoon Haiyan. It shows that mental health problems persisted beyond the typhoon's immediate aftermath among a large number of survivors. Since the mental health system was fragile, the affected community improved their mental health services through the help of local and international non-governmental organisations. Nonetheless, several challenges must be addressed as the country faces the climate crisis.

Climate change has a negative impact on the mental health of populations. Climate-related changes in humidity, rainfall, droughts, wildfires and floods are associated with psychological distress, poorer mental health, increased mortality among people with mental disorders, higher psychiatric hospital admissions and heightened suicide rates.^1^ In the Philippines, evidence suggests that climate-related events may worsen anxiety, distress and health inequalities among Filipinos.^2,3^

## Climate change in the Philippines

The Philippines is one of the world's most climate-vulnerable countries.^2^ It is confronted with at least 20 typhoons every year, which lead to the destruction of houses and livelihoods, displacement of thousands and hundreds of deaths.^3^ It also experiences extreme droughts and rising sea levels.^2,3^ These not only lead to the forced displacement of communities but also threaten food security.^2,3^ Given the negative effects of these adverse social and environmental conditions on mental health,^4^ climate-related anxiety has affected the Philippine population. In 2022, a global survey showed that the Philippines has the highest number of young people experiencing high levels of anxiety and negative emotions associated with the climate crisis.^3^ Thus, there is an ever-increasing need for a strong and resilient mental health system.

## Typhoon Haiyan and its mental health repercussions

The intersection of mental illness and the climate crisis in the Philippines is exemplified in the aftermath of Super Typhoon Haiyan (local name Yolanda) in November 2013.^5,6^ Typhoon Haiyan is one of the strongest typhoons to ever hit land in recorded history.^5,6^ It has turned thriving communities into wastelands, destroyed decades-long livelihoods, displaced four million people, destroyed one million homes and killed at least 6000 people.^5,7^ In its aftermath, local health authorities noted that those needing psychological help easily tripled because the population ‘were all shocked’.^5^ Likewise, the authorities also stated that there were rising cases of post-traumatic stress disorder (PTSD) and depression.^5^ Although no official records were collated at that time owing to limited resources and lack of structure,^5^ a study found that about 80.5% of survivors who helped with the typhoon relief response were at risk for mental disorders 4 months after the typhoon.^8^ This rate of people at risk for mental disorder following the typhoon is higher than the estimated national rate of common mental disorders in the Philippines, such as schizophrenia (0.4%) and depression (14.5%), before the typhoon.^9^ It is also higher than the rate of PTSD (7–24%) found after other natural disasters, such as the 2005 Hurricane Katrina, the 2004 Asian tsunami and the 2010 Yushu earthquake, as well as depression (14%) and psychological distress (15%) following the 2011 Japan earthquake.^10^

However, at that time, only ten psychiatrists served a population of 4.7 million in the Philippines’ critically hit region, Eastern Visayas.^5^ This was worsened by the badly damaged regional hospital.^5,6^ Thus, international and national organisations became the primary providers of mental health services.^5^ However, it was reported that there was no clear structure in these services: ‘some were already “overprocessed,” having received so many psychological services. But many others, especially poorer communities, were left behind’.^5^ Thus, the months following the aftermath of Typhoon Haiyan led to extreme social and mental health adversities against a background of a fragile mental health system.

A year after, the World Health Organization (WHO) estimated that over 800 000 people in the region suffered from various mental health conditions. Although most of these people could be treated in their homes, at least 10% needed comprehensive psychiatric treatment for depression, anxiety, PTSD or schizophrenia, including further medication and support.^6^ For the survivors, it was ‘stark evidence [of] why we need to address the mental health situation, because every time these disasters come, it takes a mentally healthy individual to cope with challenges’.^5^ Hence, mental health adversities from Typhoon Haiyan persisted in a large number of survivors even a year later.

Given the evident shortcomings in the mental health care system and the persistent mental health problems after Typhoon Haiyan, the Eastern Visayas region partnered with the WHO to establish the Mental Health Gap Action Programme for all its health units.^5^ As a result, by December 2014, it became the first Philippine region where mental healthcare and support are present at primary, secondary and tertiary healthcare levels.^5^ In the same year, 300 community workers and 70 health professionals were trained to assess and treat severe mental health problems.^5^ The regional government further supported these efforts by allocating a budget for disaster response and community-based interventions for psychosocial needs in the succeeding years.^5^ Thus, various mental health system improvements have been accomplished in Eastern Visayas by incorporating mental health services into primary care and augmenting the mental health workforce with the help of non-governmental organisations.

## Towards building a climate-responsive mental health system

Although several improvements have indeed been accomplished, several challenges remain for the Philippine mental health system. Among other things, mental health stigma remains pervasive in the Philippines, including Eastern Visayas.^5^ Moreover, only 3–5% of the total government health budget is spent on mental health.^2^ Human resources are also scarce, with only about 0.5 psychiatrists per 100 000 population and a total ratio of 2 to 3 mental health workers per 100 000 population.^11,12^ Given this situation, the country's new Mental Health Act in 2018 was met with the hope of filling these gaps in mental health services.^2^

The Mental Health Act mandates the government to strengthen mental health research, increase the mental health workforce through training, provide mental health services in all hospitals and community health centres, and expand mental health promotion in schools, communities and workplaces.^2,11,12^ In addition, envisioning the future climate crisis needs of the Philippines, it advocates hope that it can act as a springboard for creating a climate-resilient and accessible mental health system.^2^

Nonetheless, the Mental Health Act has been critiqued as ‘nothing more than “just an act”’.^13^ This is because healthcare expenditure for mental health remained at 3–5% despite the Act's implementation.^13,14^ Likewise, the ratio of psychiatrists remained low (0.4 psychiatrist per 200 000 population) compared with other Western Pacific countries of similar economic status, such as Indonesia.^13^ Moreover, there remains a paucity of research that can translate to evidence-based culturally sensitive interventions and policies.^14^ The Act's implementation was also criticised for its disproportionate focus on clinical mental health, resilience and individual coping, despite the resonating need for social intervention for social environmental factors, such as climate change, experienced in the Philippines.^14^ Consequently, mental health promotion and services were regarded as outdated despite the new Mental Health Act.^13,14^

Given the Philippines’ vulnerability to the worsening climate crisis and the weaknesses in its mental health system, reforms and improvements are needed in mental health services, resources and policy implementation. As exemplified by the Eastern Visayas region, this can be started by integrating mental health services into primary care services, increasing the mental health training of health professionals and community workers, collaborating with non-governmental organisations and sustaining support towards achieving better mental health.^5,6^ In addition, the low number of psychiatrists and mental health professionals can be addressed by supporting and increasing training institutions and their capacity. Furthermore, since there is high climate-related anxiety among Filipino youth, hope-based climate education for the empowerment of young people can also be provided since there is evidence that this might help them cope.^7^ Likewise, mental health information systems need to be strengthened to adequately assess the needs of disaster-affected localities, including accounting for affected populations and mental health workers. By doing so, data from the information systems can inform mental health and psychosocial support services in affected localities. Importantly, the implementation of the Mental Health Act must be strengthened.

## Conclusions

Overall, the mental health repercussions of the climate crisis are experienced annually in the Philippines. It has exposed weaknesses in the Philippines’ mental health system, including low human resources, lack of funding and poor policy implementation. Despite having limited resources, as have other low- and middle-income countries, societal efforts paved the way for improvements in recent years. Nonetheless, more needs to be done as the country gears up for future challenges stemming from the climate crisis. As a start, evidence suggests a need to strengthen disaster responder training, social support, surveillance systems and communication in disaster response.^8,10^ Moreover, further research, including longitudinal studies, is needed to understand climate-related mental health conditions and responses.^2,8,10^

## Data Availability

Data availability is not applicable to this article as no new data were created or analysed in this study.
